# *Krameria lappacea* roots extract to rescue coccidiosis-mediated inflammation in the jejunum of C57BL/6 mice

**DOI:** 10.3389/fimmu.2025.1557235

**Published:** 2025-04-17

**Authors:** Rewaida Abdel-Gaber, Ghada Alamari, Mohamed A. Dkhil, Saeed El-Ashram, Nawal Al-Hoshani, Esam M. Al-Shaebi, Saleh Al-Quraishy

**Affiliations:** ^1^ Department of Zoology, College of Science, King Saud University, Riyadh, Saudi Arabia; ^2^ Department of Zoology and Entomology, Faculty of Science, Helwan University, Cairo, Egypt; ^3^ Applied Science Research Center, Applied Science Private University, Amman, Jordan; ^4^ College of Life Science and Engineering, Foshan University, Foshan, Guangdong, China; ^5^ Faculty of Science, Kafrelsheikh University, Kafr El-Sheikh, Egypt; ^6^ Department of Biology, College of Science, Princess Nourah bint Abdulrahman University, Riyadh, Saudi Arabia

**Keywords:** murine coccidiosis, natural plants, *Krameria lappacea*, inflammation, immune response

## Abstract

**Introduction:**

Coccidiosis is a protozoan disease caused by *Eimeria* species, which multiply in the intestinal tract and lead to severe inflammatory responses. While coccidiostats are available for control, resistance to these treatments has been confirmed, underscoring the need for new eco-friendly approaches. In recent years, natural plant sources have gained attention as effective alternatives for treating various parasitic diseases. *Krameria lappacea* has been used in traditional medicine due to its pharmacological properties. This study examined the effects of the aqueous methanolic extract of *K. lappacea* roots (KLRE) on jejunal inflammation and immune response in a murine model infected with *Eimeria papillata*.

**Methods:**

Twenty-five male C57BL/6 mice were randomly divided into five groups. The first group received only distilled water, while the second group was administered 200 mg/kg of KLRE for 5 days. The third, fourth, and fifth groups were orally injected with 10^3^ sporulated oocysts of the *Eimeria* parasite. For treatment, the fourth group received KLRE (200 mg/kg), and the fifth group received amprolium (120 mg/kg) orally for 5 days. All mice were euthanized on day 5 post-infection (p.i.), and blood samples and jejunum were collected. Investigations were conducted to assess oocyst shedding, cellular immune response, and the histological changes in the jejunum of the mice. Levels of interleukin (IL)-1β, IL-6, and tumor necrosis factor (TNF)-α were measured using an enzyme-linked immunosorbent assay (ELISA). Additionally, the mRNA expression of CXC motif chemokine ligand 10 (CXCL10), interferon-inducible gene 202b (IFi202b), and secreted phosphoprotein 1 (SPP-1) was analyzed using quantitative reverse transcription polymerase chain reaction (qRT-PCR).

**Results:**

Our study demonstrated that mice infected with *E. papillata* produced an average of 5.387 × 10^6^ ± 4.29 × 10^5^ oocysts per gram of feces by day 5 post-infection. In contrast, the output was significantly reduced to 1.308 × 10^6^ ± 1.36 × 10^5^ oocysts per gram of feces in mice treated with KLRE. These findings suggest that the host immune response to the intracellular *Eimeria* parasite triggers inflammation and injury in the jejunum of the mice. This was evidenced by several factors: (i) an elevated inflammatory histological score, (ii) an increased cellular immune response characterized by neutrophils and lymphocytes, (iii) elevated protein levels of IL-1β, IL-6, and TNF-α, measured at approximately 13.67 ± 2.07, 78.98 ± 4.17, and 222.28 ± 10.18 pg/ml, respectively, and (iv) upregulated expression of the mRNA genes CXCL10, IFi202b, and SPP-1, which showed fold changes of approximately 2.83, 3.55, and 3.07-fold, respectively. Our study found that all parameters associated with the infection were significantly altered during treatment with KLRE.

**Conclusion:**

Our data showed that KLRE treatment significantly reduced inflammation and histological damage in the jejunum caused by *E. papillata* infections.

## Introduction

Coccidiosis is a widespread and infectious disease caused by a group of microscopic, single-celled organisms known as *Eimeria*, which belong to the apicomplexan family Eimeriidae. This parasitic disease affects a variety of vertebrates, including livestock and poultry, and can lead to serious health issues in infected animals. The impact of coccidiosis is significant, often resulting in considerable economic losses for farmers and the agricultural industry as a whole (1). *Eimeria* species strongly prefer specific hosts and sites (2). *Eimeria papillata* is one of the various *Eimeria* species that infect the mouse jejunum, resulting in significant damage to the intestinal mucosa, oxidative stress, and inflammation that affects general body performance (3).

Infection starts when sporulated *Eimeria* oocysts are ingested from the environment. Once in the intestine, these oocysts release infectious sporozoites. The sporozoites primarily invade the intestinal epithelial cells, where they multiply asexually. Eventually, the oocysts are expelled with the feces (4). The monoxenous life cycle of *Eimeria* involves various stages, including intracellular, extracellular, asexual, and sexual phases. As a result, immune responses to *Eimeria* are complex and engage multiple aspects of nonspecific and specific immunity (cellular and humoral immune mechanism) (5–7). The lymphocytes, macrophages, and other effector cells secrete cytokines and chemokines, targeting the appropriate immune responses to the invading *Eimeria* parasite (8, 9).

Anticoccidial medications, such as nicarbazin, sulfaquinoxaline, amprolium, toltrazuril, and nitrofurazone, play a crucial role in managing coccidiosis, a parasitic disease that affects various livestock. These drugs are typically administered over extended durations through drinking water and feed, which allows for consistent treatment. However, this prolonged usage raises significant concerns within animal production. Firstly, it can negatively impact the immune system of the animals, making them more susceptible to infections and other health issues, as highlighted by Yunus et al. (10). Secondly, there is a growing risk of developing drug-resistant strains of the *Eimeria* parasite, a challenge documented by Abbas et al. (11), which could render these treatments ineffective over time. Moreover, the continued use of anticoccidials may lead to toxic effects on the animals’ overall health, as reported by Nogueira et al. (12). There’s also the pressing issue of harmful drug residues accumulating in animal products, raising food safety concerns for consumers, as noted by Williams (13). In light of these limitations associated with traditional coccidiostats, there has been a marked increase in research exploring alternative methods for controlling coccidiosis. This shift has led to a renewed interest in herbal treatments, which have demonstrated promising effectiveness in alleviating coccidiosis symptoms, according to findings by Abdel-Tawab et al. (14) and Hussain et al. (15). These alternative approaches offer hope for safer, more sustainable management of this challenging parasitic disease.

*Krameria lappacea* is a member of the Krameriaceae family. The roots of this plant are widely utilized in numerous herbal formulations to treat a wide variety of diseases and body disorders (16). Many reports highlight the potential therapeutic benefits of *K. lappacea*, which include its anti-inflammatory, antioxidant, antidiabetic, anticancer, and antimicrobial properties (17–21). The dried *K. lappacea* roots are rich in various bioactive compounds, such as total phenolics, flavonoid contents, lignan derivatives, tannins, benzofuran derivatives, and oligomeric proanthocyanidins (1). These compounds are believed to contribute to the diverse biological and pharmaceutical properties of *K. lappacea*.

Recently, *K. lappacea* has received more attention due to its potential anticoccidial effects. A limited number of studies have examined the impact of *K. lappacea* in mice infected with *E. papillata* (1, 22, 23). Therefore, the current study aimed to investigate the possible anti-inflammatory activity of the *K. lappacea* root extract (KLRE), using the murine *Eimeria papillata* as a model parasite for coccidiosis.

## Materials and methods

### Plant collection and extract preparation

The roots of *Krameria lappacea* were purchased from a local market in Riyadh, Saudi Arabia. A taxonomist from the Herbarium (College of Science, King Saud University) confirmed the plant’s identity and assigned it the voucher number KSU-22958. The method described by Manikandan et al. (24) was utilized to prepare a 70% methanolic extract from the roots of *K. lappacea*, referred to as KLRE. For the *in vivo* study, KLRE was dissolved in water.

### Parasite passage

*Eimeria papillata* was used as a model coccidian parasite in laboratory mice. *E. papillata* was previously characterized in detail (25). Five laboratory mice (*Mus musculus*) were orally infected with 10^5^ sporulated oocysts via gavage to propagate the oocysts. Feces were collected five days post-infection (p.i.), and the oocysts were isolated using the flotation technique (26). The isolated oocysts were then allowed to sporulate in a 2.5% (w/v) solution of potassium dichromate (K_2_Cr_2_O_7_) before being washed in a phosphate-buffered saline (pH 7.4) (Sigma Aldrich, Taufkirchen, Germany) for use in the experimental study.

### Experimental design

The animal facility at King Saud University in Riyadh, Saudi Arabia, provided a cohort of twenty-five male C57BL/6 mice, each carefully selected for their age of 10 to 12 weeks. These mice, weighing between 20 and 25 grams, were bred under strict pathogen-free conditions to ensure their health and well-being. Each mouse enjoyed the freedom of accessing food and water *ad libitum*, promoting their natural behaviors. They were housed in spacious plastic cages designed to facilitate comfort and ventilation within a meticulously maintained, temperature-controlled environment. The facility operated on a consistent 12-hour light/dark cycle, mimicking natural conditions to support their circadian rhythms. Before the commencement of the experiment, the mice underwent a one-week acclimatization period, allowing them to adjust seamlessly to their surroundings.

**Figure 1:**
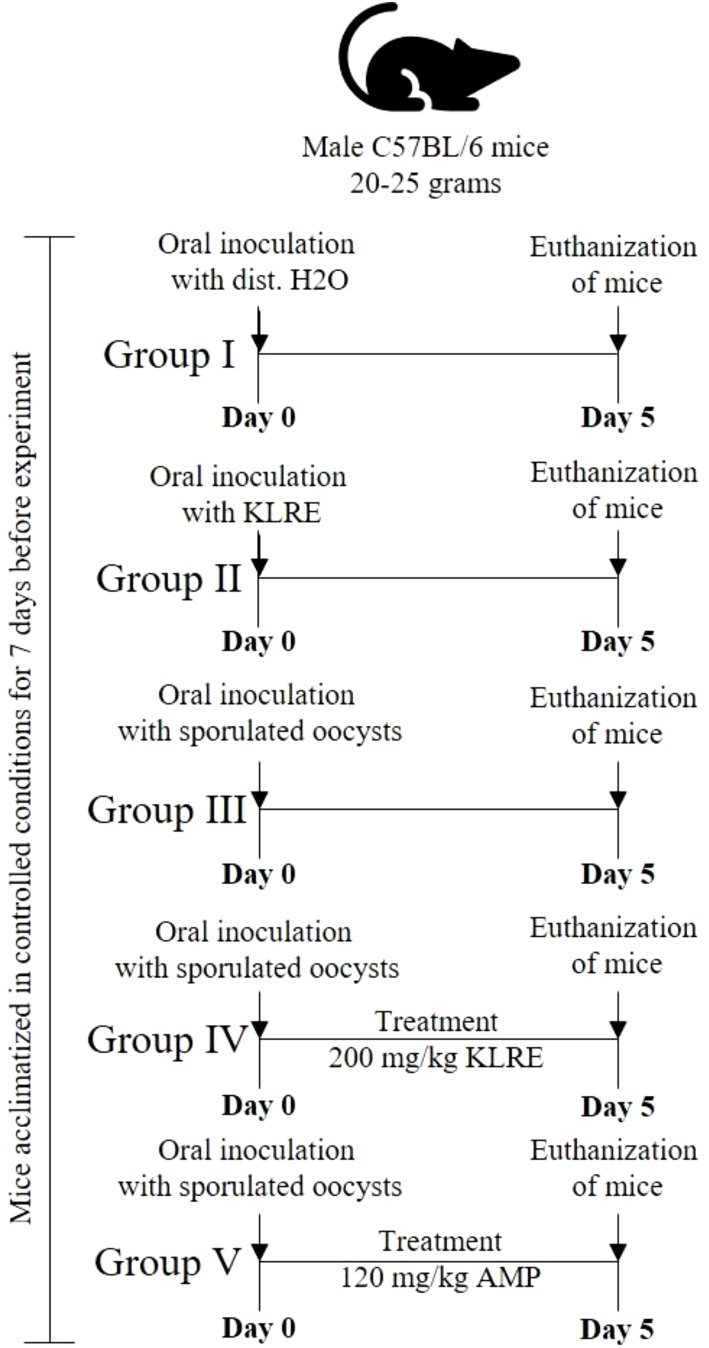
The timeline of the experiment design shows the mice groups, the inoculation of sporulated *Eimeria papillata*, and the euthanization day.

The mice were divided into five groups of five mice each (**Figure 1**), as follows:

Group I - Uninfected, untreated (negative control).
Group II - Uninfected, treated with 200 mg/kg KLRE (27).
Group III - Infected, untreated (positive control).
Group IV - Infected and treated with 200 mg/kg KLRE (27).
Group V - Infected and treated with 120 mg/kg Amprolium (28).

All experimental groups, except for groups I and II, were orally administered a suspension containing 10^3^ sporulated oocysts of *E. papillata* in 100 µl of physiological saline, as detailed by Abdel-Tawab et al. (14). Group I, serving as a control, was given distilled water instead of the parasite. Meanwhile, Group II received a solution of KLRE (presumably a treatment or supplement) dissolved in distilled water. The treatment period was maintained for five consecutive days. On the fifth day post-infection (p.i.), the shedding of *Eimeria* oocysts was quantified in fecal pellets from infected untreated and treated groups using a McMaster chamber and then expressed as the number of *Eimeria* oocysts per gram (OPG) of wet feces following the method outlined by Dkhil et al. (29).

### Samples collection

All mice were euthanized with CO_2_ asphyxiation. Blood was collected from the heart into heparinized tubes. A thin smear was prepared, stained with Giemsa, and examined under a Leica DM 2500 microscope using NIS ELEMENTS software (version 3.8) for differential cell counts. The jejuna of the mice were isolated and cut into small pieces for the following purposes: (a) buffered formalin (10%) was used for histological analysis, (b) small tubes were kept at -80°C to investigate protein expression, and (c) RNA Later^®^ (Invitrogen, Carlsbad, CA) was used for molecular analysis and stored at -80°C.

### Histological examination

The jejuna samples were fixed in phosphate-buffered formalin (10%) for 24 hr at 27°C. This preserved tissue was subsequently processed, sectioned (5 µm), and stained with hematoxylin-eosin (H&E) according to the method described by Drury and Wallington (30). The histopathological evaluation was carried out using a Leica DM 2500 microscope, complemented by the NIS ELEMENTS software (version 3.8) to facilitate detailed analysis. To maintain objectivity in the assessment, all evaluations were performed in a blinded manner. The comprehensive assessment of total histological injury involved a thorough examination of various factors, including inflammatory lesions, the extent of tissue destruction, and the processes of tissue repair. This analysis was guided by the systematic criteria outlined by Dommels et al. (31), ensuring a robust and reliable interpretation of the histological findings. Each aspect was assigned a rating score from 0 to 3, where 0 indicates no change from normal tissue and 3 indicates that lesions were widespread, affecting most areas and all layers of the intestinal section. Furthermore, the total score for inflammatory lesions was multiplied by 2 to emphasize this value, especially when these lesions mainly characterized the tissue changes.

### Sandwich enzyme-linked immunosorbent assay

Quantitative measurements of interleukin (IL)-1β (Cat no. ab197742, Abcam, USA), IL-6 (Cat no. MBS730957, MyBioSource, USA), and tumor necrosis factor (TNF)-α (Cat no. SEK50349, Sino Biological, USA) levels were performed using ELISA kits specified for mice according to the protocol provided with each kit. Optical densities (OD) of outcomes from the jejunal samples were measured using the Bio-Rad IMark Microplate Reader SW 1.04.02.E. Based on a standard curve, OD values were converted to concentrations and presented as pg/ml.

### RNA extraction and qRT-PCR

Total RNA was extracted from jejunal tissues fixed in RNAlater using the QIAamp RNeasy Mini Kit (Qiagen Inc., Valencia, CA). The concentration and purity of each RNA sample were assessed at an A_260/280_ ratio using a NanoDrop ND-1000 Spectrophotometer (NanoDrop Technologies, Wilmington, Delaware, USA) and stored at -80 °C until use. To remove contaminating DNA, RNA samples were treated with DNase (Applied Biosystems, Darmstadt, Germany). The RNA was then stored at -80°C until reverse transcribed into cDNA using the miScript™ Reverse Transcription Kit (Qiagen, Hilden, Germany) following the manufacturer’s instructions and stored at -20 °C.

For the real-time polymerase chain reaction (RT-PCR) analysis, cDNA samples were tested in triplicate. The PCR amplification included non-template controls, containing all reagents except for cDNA. Real-time quantitative PCR (qPCR) was performed using the ABI Prism 7500HT Sequence Detection System (Applied Biosystems, Darmstadt, Germany) with Thermo Scientific Maxima^®^ SYBR Green PCR Master Mix (2×) (Thermo Fisher Scientific Inc., California, USA). The PCR process began with an initial incubation at 50 °C for 2 min, followed by activation of Taq polymerase at 95 °C for 15 min. This was followed by 40 cycles of 95 °C for 15 sec, 50 °C for 35 sec, and 70 °C for 30 sec.

As shown in **Table 1**, PCR primers for the genes of CXC motif chemokine ligand 10 (CXCL10), interferon-inducible gene 202b (IFi202b), and secreted phosphoprotein 1 (SPP-1) were synthesized using Primer Express 3.0 (Applied Biosystems, USA) and obtained commercially from Qiagen (Hilden, Germany). All PCR reactions yielded only a product of the expected size detected by melting point analysis and gel electrophoresis. The amplification data were quantitatively evaluated using the TaqMan 7500 system software version 1.2.3f2 (Applied Biosystems, Darmstadt, Germany) and analyzed with the Ct method (2^−ΔΔCT^) as described by Livak and Schmittgen (32). β-actin gene was used as the housekeeping gene for data normalization.

**Table 1:** Primers used for real-time PCR analysis of genes involved in this study.

Genes	Biological classification	Primer direction	Primer sequence (5’ → 3’)
CXC motif chemokine ligand 10 (CXCL10)	Cell-to-cell signaling and interaction	Forward	5’- ATCATCCCTGCGAGCCTATCCT -3’
		Reverse	5’- GACCTTTTTTGGCTAAACGCTTTC -3’
Interferon-inducible gene 202b (IFi202b)	Cellular growth and proliferation	Forward	5’- CCGGGAAACACCATTGCTT -3’
		Reverse	5’- ACCTCAGACACGCTGGAATATTC -3’
Secreted phosphoprotein 1 (SPP-1)	Inflammatory response	Forward	5’- GCTTGGCTTATGGACTGAGGTC -3’
		Reverse	5’- CCTTAGACTCACCGCTCTTCATG -3’
Beta-actin (β-actin)	Housekeeping gene	Forward	5’- GCTACAGCTTCACCACCACA -3’
		Reverse	5’- AAGAAGGCTGGAAAAGAGC -3’

### Statistical analysis

The collected data underwent thorough analysis using Tukey *post hoc* analysis, with a significance level set at a p-value of 0.05 or lower. The results were presented as means accompanied by standard deviations (SD) to provide a clear understanding of the variability within the data. For this analytical process, SigmaPlot^®^ version 11.0, developed by Systat Software, Inc. based in Chicago, IL, USA, was utilized to facilitate the statistical evaluations.

## Results

All mice that were orally inoculated with 10^3^ sporulated *E. papillata* oocysts began shedding oocysts after three days post-inoculation (p.i.). As shown in **Table 2**, the shedding of *E. papillata* oocysts reached its peak on the 5^th^-day p.i. The inoculation of KLRE significantly reduced the number of oocysts excreted in feces, decreasing the count from 5.387 × 10^6^ ± 4.29 × 10^5^ to 1.308 × 10^6^ ± 1.36 × 10^5^ oocysts per gram of feces. Additionally, KLRE demonstrated maximum anti-coccidial effectiveness, outperforming amprolium, which resulted in 1.850 × 10^6^ ± 6.04 × 10^5^ oocysts per gram of feces (**Table 2**).

**Table 2:** KLRE-induced reduction in oocyst output.

Experimental group	Oocyst output/g feces
Uninfected + untreated	–
Uninfected + treated KLRE	–
Infected + untreated	5.387 × 10^6^ ± 4.29 × 10^5^
Infected + treated KLRE	1.308 × 10^6^ ± 1.36 × 10^5 #^
Infected + treated AMP	1.850 × 10^6^ ± 6.04 × 10^5 #^

^#^ indicates significant difference against infected group (p ≤ 0.05), data are represented as mean ± standard deviation (SD).

**Figure 2:**
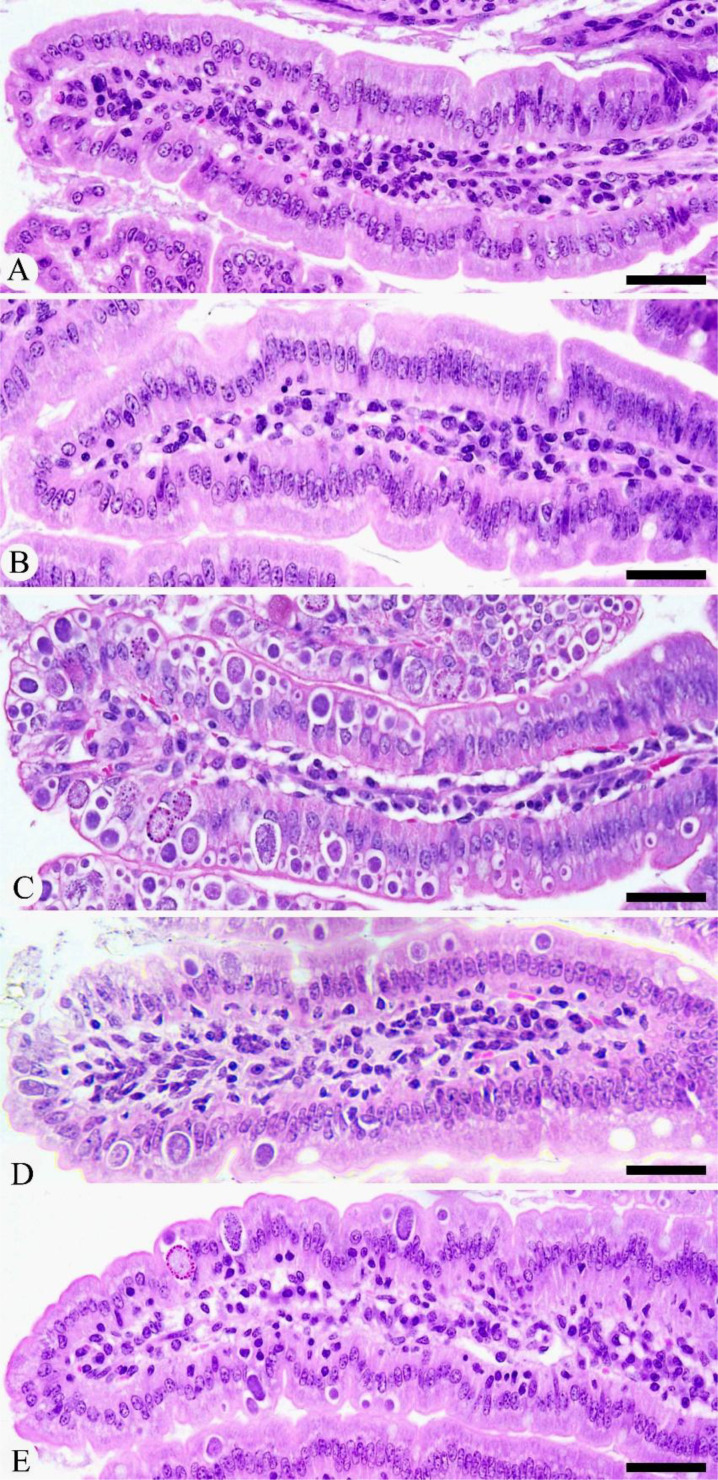
Histological changes during *Eimeria* infection and after treatment with KLRE. **(A)** Control jejunum. **(B)** KLRE-treated jejunum. **(C)** Infected jejunum. **(D)** Infected-treated jejunum with KLRE. **(E)** Infected-treated jejunum with AMP. Scale bar = 100µm.

**Figure 3:**
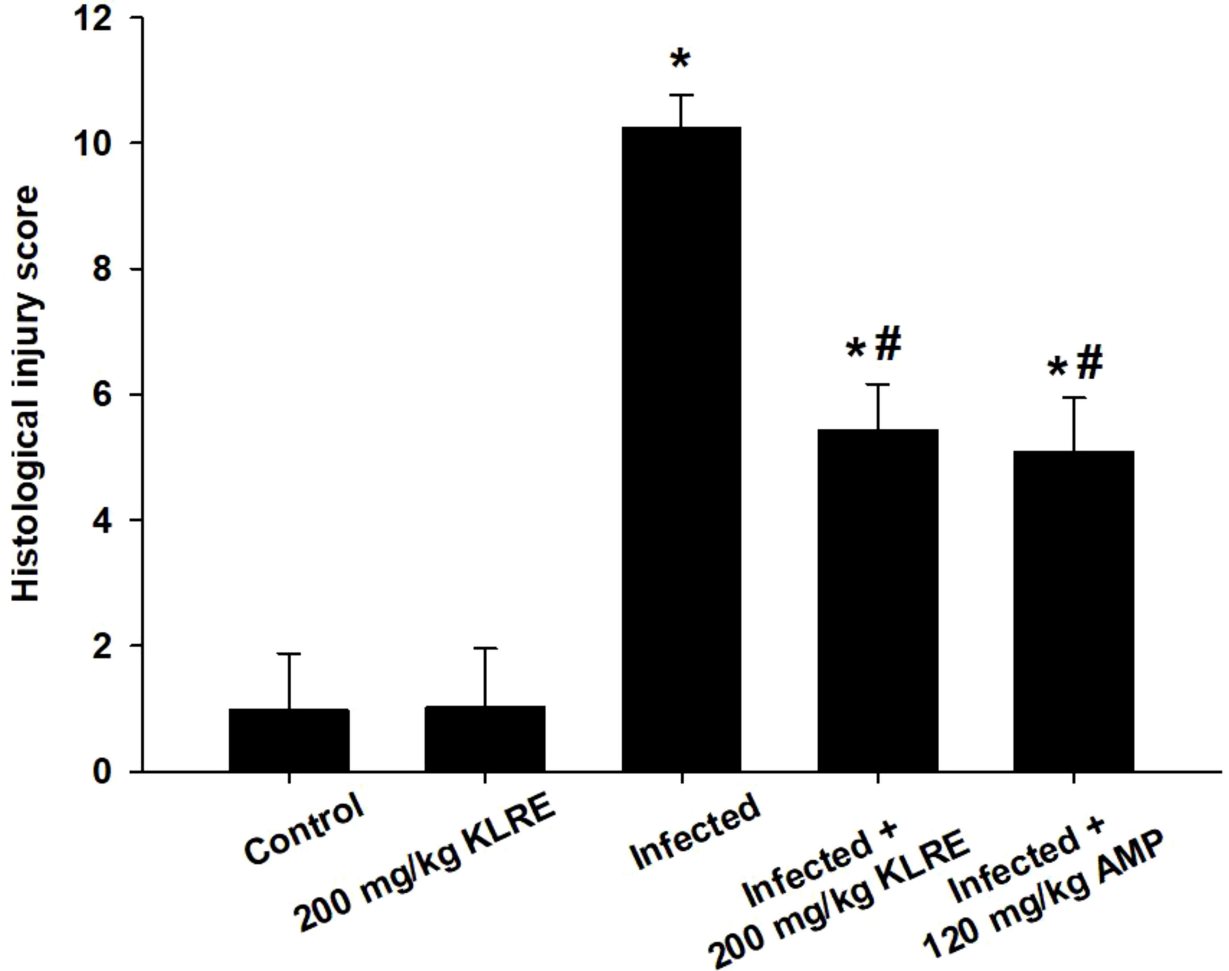
The score of histological injury of the experimental mice groups. ^*,#^ indicates significant difference against control and infected groups, respectively (p ≤ 0.05). Data are represented as mean ± standard deviation (SD).

Experimental infection with *E. papillata* oocysts led to significant histological changes in the jejunal mice tissues, with various parasite developmental stages evident within the parasitophorous vacuoles in the epithelial intestinal cells (**Figure 2**). Moreover, the infected jejunal tissues exhibited notable signs of necrotic enteritis, alongside a moderate degree of inflammatory damage attributed to the various stages of *Eimeria* infection (**Figure 3**). According to the analysis conducted by Dommels et al. (31), the administration of KLRE in conjunction with amprolium proved effective in alleviating symptoms of enteritis. This combined treatment not only significantly mitigated the levels of necrosis and inflammation observed in the jejunal tissues of the mice, but it also corresponded with a marked reduction in the count of *Eimeria* stages present, as illustrated in **Figures 2**, **3**.

The effect of KLRE on white blood cells (WBCs) in mice infected with *Eimeria* oocysts is detailed in **Table 3**. *Eimeria* infection caused a significant inflammatory response, increasing neutrophils (39.40 ± 1.06) and lymphocytes (59.05 ± 1.51) in the infected group compared to the control and uninfected-treated KLRE groups. KLRE treatment significantly improved neutrophil and lymphocyte levels compared to the infected group (**Table 3**).

**Table 3:** KLRE ameliorates *E. papillata*-induced changes in neutrophils and lymphocytes.

Experimental group	Neutrophils (%)	Lymphocytes (%)
Uninfected + untreated	20.56 ± 1.12	29.36 ± 1.85
Uninfected + treated KLRE	21.25 ± 1.54	30.12 ± 1.01
Infected + untreated	39.40 ± 1.06 ^*^	59.05 ± 1.51 ^*^
Infected + treated KLRE	28.05 ± 1.32 ^*#^	38.71 ± 1.32 ^*#^
Infected + treated AMP	27.29 ± 1.72 ^*#^	42.92 ± 1.92 ^*#^

^*^,^#^ indicates significant difference against control and infected groups, respectively (p ≤ 0.05). Data are represented as mean ± standard deviation (SD).

**Figure 4:**
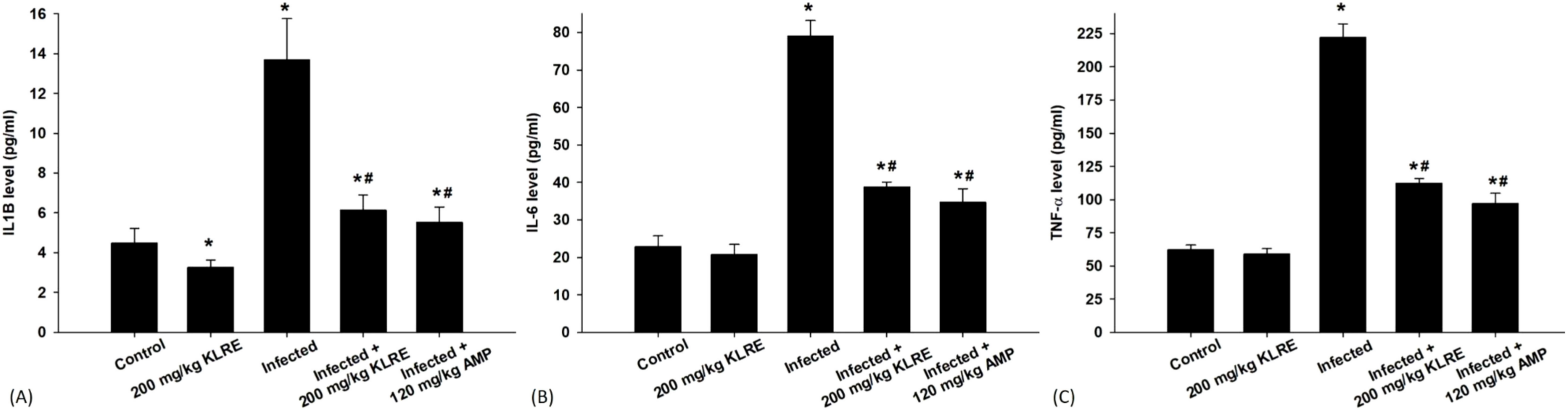
Levels of IL1β **(A)**, IL-6 **(B)**, and TNF-α **(C)** in the jejunal samples from the different experimental mice groups. ^*,#^ indicates significant difference against control and infected groups, respectively (p ≤ 0.05). Data are represented as mean ± standard deviation (SD).

The ELISA method was utilized to assess changes in the protein levels of inflammatory cytokines in jejunal tissue samples from mice (**Figure 4**). *Eimeria* infection significantly increased IL-1β, IL-6, and TNF-α levels compared to their non-infected counterparts. Specifically, the protein levels rose to approximately 13.67 ± 2.07, 78.98 ± 4.17, and 222.28 ± 10.18 pg/ml, respectively, compared to the control group (**Figure 4**). In contrast, treatment with KLRE resulted in a notable downregulation of these proteins, with levels decreasing to about 6.14 ± 0.76, 38.85 ± 1.24, and 112.41 ± 3.60 pg/ml, respectively (**Figure 4**).

**Figure 5:**
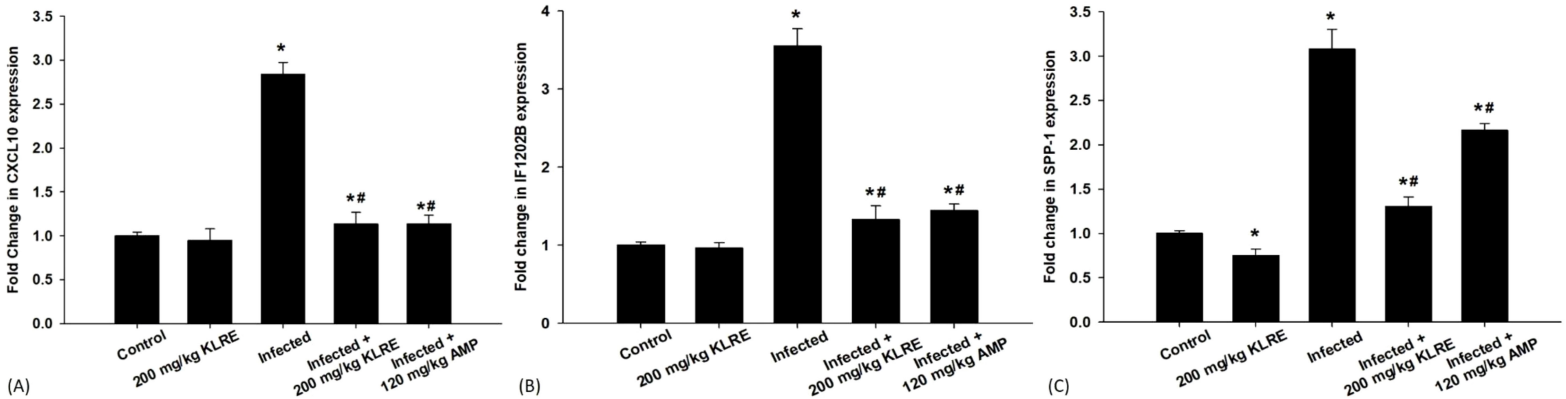
mRNA expression of CXCL10 **(A)**, IF1202B **(B)**, and SPP-1 **(C)** in the jejunal samples from the different experimental groups. The expression values obtained by RT-PCR analysis were normalized to the reference gene GAPDH mRNA level and are shown as fold induction (in log 2 scale) relative to the mRNA level in the control. ^*,#^ indicates significant difference against control and infected groups, respectively (p ≤ 0.05). Data are represented as mean ± standard deviation (SD).

The mRNA expression of the CXCL10 gene significantly increased by approximately 2.83-fold in the *E. papillata*-infected group compared to controls (**Figure 5A**). This gene is released in response to high IFN-γ levels from CD4 cells. Treatment with KLRE resulted in a downregulation of CXCL10 by about 1.133-fold, similar to the reference drug’s 1.138-fold reduction (**Figure 5A**). Moreover, qRT-PCR results showed that IFi202B gene expression increased in the jejunum of the infected mice after growth and proliferation of the *Eimeria* stages within the epithelial cells, leading to adipocyte hypertrophy, lipid accumulation, and higher malondialdehyde levels (**Figure 5B**). KLRE treatment reduced IFi202B mRNA expression from 3.55-fold to 1.32-fold, suggesting improved cellular conditions (**Figure 5B**). *Eimeria* infection in mice caused a 3.07-fold increase in SPP-1 mRNA expression compared to the control group, indicating its role in immune regulation after *Eimeria* infection, with SPP1 potentially interacting with receptors that aid *Eimeria* invasion (**Figure 5C**). Treatment with KLRE significantly reduced this expression to 1.30-fold, lower than the reference drug’s level of 2.16-fold (**Figure 5C**).

## Discussion

*Eimeria* is one of the leading causes of morbidity and mortality in various animal species (22). Previous studies have examined intestinal infection sites and the use of natural resources as alternatives to coccidiostats for treating coccidiosis in mice (33). Natural remedies employ bioactive compounds derived from plants to specifically target parasitic infections, achieving therapeutic efficacy while minimizing the adverse effects commonly associated with synthetic pharmaceuticals (34, 35). In Saudi Arabia, a range of medicinal plant extracts has demonstrated significant anti-eimerial activity. Notable among these are *Ziziphus* sp*ina-christi*, known for its resilience in arid environments and its traditional use in folk medicine (36); *Glycyrrhiza glabra*, commonly called licorice, which has been valued for its therapeutic properties (37); *Salvadora persica*, also known as the toothbrush tree, recognized for its dental benefits (38); *Astragalus membranaceus*, a herb celebrated in traditional Chinese medicine for its immune-boosting abilities (14); and *Azadirachta indica*, or neem, renowned for its wide array of health benefits and pest-repelling properties (39). These plants showcase the rich biodiversity and the potential of natural remedies in combating specific health challenges. KLRE has shown promise against various diseases and disorders (17–21). Additionally, our previous studies demonstrated the anti-coccidial activity of this plant extract against the *E. papillata* murine model (1, 22, 23). This study investigates inflammatory responses and immune activities in the jejunum of mice after *Eimeria* infection and the role of KLRE in modulating these responses.

Our findings show that KLRE has anti-coccidial activity, reducing *E. papillata* oocysts in the feces of infected mice by day 5 post-infection. This reduction suggests reduced parasite development and expulsion. KLRE disrupts the oocyst membrane, affecting cellular permeability and leading to the death of *Eimeria* stages. The efficacy of KLRE is attributed to the polyphenolic components significantly impacting *Eimeria* parasites, confirming its anti-coccidial properties (1, 17, 18, 20–23). With minimal adverse effects, this action is comparable to coccidiostats as amprolium, which interferes with the *Eimeria* parasite’s thiamine metabolism, effectively reducing oocyst output.

The histopathological examination demonstrated a severe infection caused by *Eimeria*, marked by the presence of various developmental stages of the parasite specifically located in the crypt region of the jejunum. This infection was associated with pronounced inflammatory damage, resulting in significant alterations to the tissue structure. These findings align closely with those documented in studies by Al-Quraishy et al. (4)Laurent et al. (6) Withanage et al. (40) Kaneko et al. (41) Dkhil et al. (42) Tomal et al. (43) Liu et al. (44) Souza et al. (45), and Su et al. (46), all of which highlight the critical impact of *Eimeria* infections on intestinal health. In addition, Tomal et al. (43) emphasize that the microbiota is essential in modulating the acute inflammatory response during *E. tenella* infection. Their research highlights how changes in the microbiota can significantly impact the body’s immune reactions, ultimately leading to a compromise in the integrity of the intestinal barrier. This interaction not only exacerbates the inflammatory response but also makes the intestine more susceptible to damage and dysfunction. During the *Eimeria* species infection in cattle, tissue damage leads to the release of pro-inflammatory cytokines, as reported by van Miert (47) and Sevimli and Kuş (48). In this study, KLRE significantly reduced the development of *E. papillata* in infected mice and injury to the jejunal tissue. The data suggests that the coccidiostatic effectiveness of KLRE can be attributed to its rich content of phenolic compounds. These compounds play a crucial role in obstructing the replication of *Eimeria* parasites within the jejunal region of the intestines. By doing so, they significantly mitigate the inflammatory damage that typically occurs as a result of the parasites’ invasion, leading to improved intestinal health. Furthermore, following Carrillo et al. (49), oleic acid is one of the bioactive components of the KLRE that serves as a significant source for the complete reconversion of malignant or infected cells into healthy intestinal cells. Similar effects on parasitic progression have been observed with various coccidiostats in previous studies (1, 36, 50, 51).

This study assessed the inflammatory response to *Eimeria* infection by evaluating leukocyte infiltration, specifically neutrophils and lymphocytes, in the jejunum. This aligns with the findings of Vervelde et al. (52) Al-Quraishy et al. (53) Amer et al. (54), and Dkhil et al. (29) who reported the critical role of neutrophils and lymphocytes within the blood during the invasion of the *Eimeria* parasite in the intestinal epithelial cells of the experimentally infected mice. Treatment of the infected mice with KLRE demonstrated a significant modulation of the leukocyte count in the mice’s jejunal tissue. The notable anti-inflammatory effects of KLRE can be attributed to the active components present in the root extract (23).

In chickens infected with *Eimeria*, intestinal inflammation is linked to macrophage and T lymphocyte infiltration as well as elevated cytokine and chemokine production (55). This aligns with the data provided in this study for the upregulation of cytokines and chemokines, which plays an important role in intestinal inflammation as a feature of the disease. We observed, herein, elevated levels of a potent pro-inflammatory cytokine, IL-1β, following infection with *E. papillata*. This finding aligns with the research conducted by Hong et al. (56), who reported an upregulation of IL-1β in response to infections with *E. acervulina* or *E. tenella*. IL-1β is a key inflammatory cytokine that is predominantly synthesized by activated neutrophils and macrophages. These immune cells travel from the bloodstream to the specific areas of infection, where they are tasked with eliminating the proliferative stages of the *Eimeria* parasite. IL-1β is essential for initiating and regulating the innate immune response, as it plays a vital role in attracting additional inflammatory cells to the site of *Eimeria* infection. This recruitment is crucial for mounting an effective defense against the invasion and proliferation of the parasite, as highlighted in research by Hong et al. (56). Our results illustrate that KLRE serves as a protective agent for jejunal tissues by exerting anti-inflammatory effects alongside targeting *Eimeria* stages. This particular action may be linked to the beneficial properties of flavonoid compounds, which have been shown to significantly lower the production of pro-inflammatory cytokines. As suggested by Baumgartner et al. (18), these compounds play a crucial role in modulating inflammatory responses in the body.

Moreover, a significant increase in IL-6 and TNF-α indicates a strong systemic inflammatory response against intracellular protozoan *E. papillata* infections in the mice model. While pro-inflammatory cytokines, such as IL-6 and TNF-α, usually play a crucial role in regulating the production of mucins, which are important components of mucus secretion. However, this study revealed that infection with *Eimeria* resulted in elevated levels of these cytokines, suggesting that the infection may significantly alter the normal regulatory mechanisms governing mucin synthesis. This elevation is linked to fewer goblet cells, as noted by Al-Quraishy et al. (23). This is consistent with the suggestion of Laurent et al. (6)Kim et al. (7) Lillehoj and Trout (57), and Tomal et al. (43), who stated that macrophages may largely participate in the pro-inflammatory response described in cecal tissues during *E. tenella* infection. Research conducted by Swaggerty et al. (58) and Hong et al. (56) highlights that an elevated expression of IL-6 plays a crucial role in enhancing the capabilities of neutrophils, which are essential components of the immune system. This increased IL-6 expression leads to a population of neutrophils that are not only more abundant but also more proficient in responding to and eliminating infections effectively. Furthermore, a body of prior research has established that TNF-α serves as the primary mediator of the inflammatory response in the body. This cytokine is pivotal in instigating a cascade of inflammatory events that significantly compromise the integrity of the intestinal barrier. The detrimental effects of these pro-inflammatory cytokines, particularly following an *Eimeria* infection, have been documented in numerous studies (43, 59–62), illustrating the complex interplay between inflammation and gut health. In addition to its ability to target multiple stages of the *Eimeria* life cycle, KLRE therapy has demonstrated a notable effectiveness in reducing the inflammatory response in infected mice. This dual action suggests that KLRE not only serves as an antiparasitic treatment but also functions as an immunomodulatory and anti-inflammatory agent. These findings align with earlier research, which highlighted the beneficial effects of KLRE as being largely attributed to its rich flavonoid content (18, 63, 64). This underscores the potential of KLRE to provide comprehensive therapeutic benefits in managing infections,

Chemokines are cytokines with chemotactic activity that act mainly by drawing leukocytes to inflammatory sites and promoting their migration from the circulation into infected tissue to mediate host defense mechanisms (65, 66). In this study, we explored adaptive immunity during *Eimeria* infection and found that the chemokine CXCL10, likely secreted by intestinal epithelial cells, was induced within the *E. papillata* infection. This response is triggered by elevated IFN-γ levels from CD4 T cells, this aligns with Abdel-Gaber et al. (22), who reported the elevation of IFN-γ during *E. papillata* infection. Previous research by Hardison et al. (67) supports this link, demonstrating that IFN-γ signaling regulates chemokine expression in *Eimeria*-infected models. CXCL10 recruits immune cells through its receptors on T lymphocytes, macrophages, and natural killer (NK) cells. Treatment with KLRE effectively controlled the infection by reducing IFN-γ levels, which inhibited CXCL10 production, highlighting its role in promoting cell-mediated immunity against parasites like *Trypanosoma cruzi* (67, 68).

After *Eimeria* infection, mRNA analysis showed significant upregulation of the IFi202B gene, linked to adipocyte hypertrophy, lipid accumulation, and increased malondialdehyde (MDA) levels. These results align with Abdel-Gaber et al. (39), indicating that *Eimeria’s* infective stages produce free radicals and reactive oxygen species (ROS), stimulating chemokine secretion and promoting inflammation. Research by Dinesh et al. (69) and Gong et al. (70) suggests a role for IFi202B in CD8 T cell suppression. Moreover, KLRE exhibits significant anti-inflammatory properties that contribute to a reduction in the severity of infections in mouse models. This observation aligns with the research conducted by Naikwadi et al. (71) Zhang et al. (72), and Al-Quraishy et al. (23), which highlights the therapeutic potential of beneficial compounds such as β- and γ-sitosterol. These compounds play a crucial role in the treatment of a variety of inflammatory diseases, further underscoring the importance of KLRE as a promising candidate in anti-inflammatory therapies.

The findings of this study reveal, for the first time, a significant upregulation of the SPP1 gene, which plays a crucial role in immune regulation, following an infection with *Eimeria*. This discovery is consistent with prior research conducted by Matsubara et al. (73) and Huang et al. (74), both of which highlighted the importance of SPP1 in the progression of diseases and the process of metastasis. Interestingly, the administration of KLRE has been shown to restore normal levels of SPP1 expression, indicating that the active components within KLRE may possess the ability to inhibit both the invasion and replication of *Eimeria* in mouse models. The beneficial effects attributed to KLRE are likely a result of its biologically active ingredients, a point further emphasized by Al-Oqail (20). This suggests not only a potential therapeutic avenue for combating *Eimeria* infections but also sheds light on the intricate interactions between immune regulation and pathogen behavior.

## Conclusion

The findings indicate that the roots of *K. lappacea* have potential as an herbal medicine due to their notable anti-inflammatory properties. These properties specifically target immune effector molecules in the jejunum, an important part of the small intestine, which are affected by infection from *E. papillata* in mouse models. This suggests that *K. lappacea* may play a crucial role in modulating immune responses during parasitic infections. However, to fully comprehend the mechanisms through which these roots exert their effects on both the *E. papillata* parasite and the host’s immune system, further research is essential. Such studies could pave the way for new therapeutic approaches utilizing *K. lappacea* in the treatment of parasitic infections and related inflammatory responses.

## Data Availability

The raw data supporting the conclusions of this article will be made available by the authors, without undue reservation.
